# The clinical features of late postoperative cholangitis following pancreaticoduodenectomy brought on by conditions other than cancer recurrence: a single-center retrospective study

**DOI:** 10.1186/s12893-022-01752-3

**Published:** 2022-08-05

**Authors:** Yasuhiro Kihara, Hiroshi Yokomizo

**Affiliations:** Division of General Surgery, Japanese Red Cross Kumamoto Hospital, Nagamineminami 2-1-1, Higashiku, Kumamoto, Kumamoto 861-8520 Japan

**Keywords:** Pancreaticoduodenectomy, Postoperative cholangitis, Tokyo Guidelines 2018

## Abstract

**Background:**

Postoperative cholangitis is a late complication of pancreaticoduodenectomy (PD). This study aimed to elucidate the pathogenesis of post-PD cholangitis (PPDC) and explore its optimal treatment.

**Methods:**

We retrospectively analyzed 210 patients who underwent PD at our institute between 2009 and 2018. Patients who underwent follow-up for less than 1 year or had cholangitis caused by cancer recurrence were excluded from the analysis. Diagnostic criteria for cholangitis and its severity were determined based on the classification of acute cholangitis provided by the 2018 Tokyo Guidelines (TG18).

**Results:**

PPDC occurred in 19 (11%) of the 176 included patients. Of these 19 patients, nine experienced more than one episode of cholangitis (total episodes, 36). For 14 patients (74%), the first episode of PPDC occurred within two years after surgery. Based on the TG18, 21 episodes were mild and 15 episodes were moderate; none were severe. Blood culture test results were positive for 16 of 24 episodes. Most patients were hospitalized and treated with intravenous antibiotics (median, seven days). The blood test values improved promptly after treatment was started. Four patients with recurrent cholangitis underwent endoscopic examination, and three of them had anastomotic stenosis of the hepaticojejunostomy. The univariate and multivariate analyses did not indicate any significant predictive factors for PPDC development.

**Conclusion:**

Mild and moderate PPDC occurred and improved with short-term antimicrobial treatment. Temporary reflux into the intrahepatic bile ducts may have been the cause of PPDC while anastomotic stenosis may be involved in recurrent cases.

## Background

Improved surgical techniques and postoperative management have enhanced the short-term postoperative outcomes of pancreaticoduodenectomy (PD) [1, 2]. As a result, long-term outcomes have become increasingly important. Postoperative cholangitis is one of the major late complications affecting long-term survivors after PD [3–5].

Common acute cholangitis, in the absence of surgical manipulation of the biliary system, is caused by stones or tumors obstructing the bile flow. Bile stasis leads to bacterial growth, and the increased pressure in the biliary tract allows bacteria and inflammatory substances to enter the veins, causing a systemic inflammatory reaction, such as sepsis. If not treated appropriately, common acute cholangitis can be fatal.

In contrast, post-PD cholangitis (PPDC) is suspected to be caused by digestive fluid reflux into the intrahepatic bile duct (IHBD), and bile congestion is attributable to anastomotic stricture [3–5]. Resection of the distal bile duct, including the sphincter of Oddi, in PD, may weaken the anti-reflux mechanism and increase the risk of digestive juice reflux into the IHBD. Additionally, anastomosis of hepaticojejunostomy may result in stenosis, which could cause bile stasis [3–5]. These findings suggest that cholangitis is more likely to occur in the post-PD state.

Although guidelines, such as the Tokyo Guidelines (TG), have been well established for common acute cholangitis, there are no specific guidelines for PPDC. Currently, the diagnosis and treatment of PPDC are based on the guidelines for common acute cholangitis. However, the anatomy of the biliary system is modified by surgery; therefore, the clinical course may be different from that of common acute cholangitis.

This study aimed to elucidate the pathogenesis of post-PD cholangitis by examining its clinical features and explore its optimal treatment. These results have not been previously described in detail.

## Methods

### Patients

**Fig. 1:**
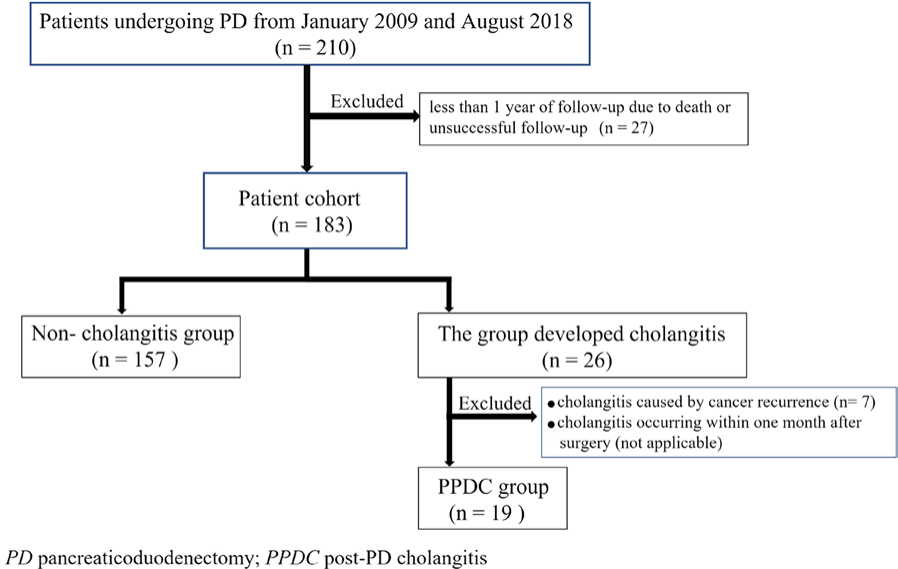
Patient enrollment flowchart

In this study, we enrolled patients who underwent PD at our institution between January 2009 and August 2018. Preoperative and postoperative clinical data were retrospectively collected. The Clinical Ethics Committee of our institute approved this study (permission no. 374), and the requirement for informed consent was waived due to the retrospective design of the study. We excluded patients from the analysis if they were followed up for less than one year due to death or unsuccessful follow-up (27 patients). In addition, we excluded cases of postoperative cholangitis caused by cancer recurrence (7 patients) and those with postoperative cholangitis that developed within one month after surgery (not applicable). Finally, 157 patients in the group without PPDC and 19 patients in the group with PPDC were included in the analysis (Fig. 1).

### Diagnostic criteria for and severity classification of postoperative cholangitis

Based on literature review and expert consensus at the International Consensus Conference on the Management of Acute Cholangitis and Cholecystitis, diagnostic criteria for common acute cholangitis were first defined in 2007 [6]. The TG were revised in 2013 [7, 8] and 2018 [9, 10]. Therefore, we evaluated PPDC according to the criteria for common acute cholangitis proposed by the 2018 Tokyo Guidelines (TG18). The diagnostic criteria and severity classifications are listed in Table 1. In this study, suspected diagnoses based on the TG18 were also classified as cholangitis.

**Table 1:** Diagnosis criteria and severity classification of PPDC based on TG18

A. Systemic inflammation		
A-1. Fever and/or shaking chills		
A-2. Laboratory data: evidence of inflammatory response		
B. Cholestasis		
B-1. Jaundice		
B-2. Laboratory data: abnormal liver function tests		
C. Imaging		
C-1. Biliary dilatation		
C-2. Evidence of the etiology on imaging (stricture, stone, stent etc		
*Suspected diagnosis: one item in A* + *one item in either B or C*		
*Definite diagnosis: one item in A, one item in B and one item in C*		
*Thresholds:*		
A-1	Fever	BT > 38 °C
A-2	Evidence of inflammatory response	WBC counts: < 4000 or > 10,000 /μL CRP: > 1.0 mg/dL
B-1	Jaudice	T-bil: > 2.0 mg/dL
B-2	Abnormal liver function	ALP, γ-GTP, AST, ALT: > 1.5 times the institutional standard values

Severity classification and treatment		
Severity	General condition	Diagnosis criteria
Mild	Not sick	Fever with mild laboratory abnormalities (cases that do not meet the criteria for moderate or severe cholangitis)
Moderate	A little tight	Presenting with two of the following events: Age ≥ 75 BT ≥ 39 ºC WBC count < 4000 or 12,000 T-bil ≥ 5 mg/dl Serum albumin level 0.73 × the standard value
Severe	Poor	Abnormalities suggestive of sepsis (associated with impairment of circulation, central nervous system, respiration, renal function, liver function, or coagulation)

*PPDC* post pancreaticoduodenectomy cholangitis, *TG18* Tokyo Guideline 2018, *BT* body temperature, *WBC* white blood cells, *CRP* C-reactive protein, *T-bil* Total bilirubin, *ALP* alkaline phosphate, *γ-GTP* gamma-glutamyl transpeptidase, *AST* aspartate aminotransaminase, *ALT* alanine aminotransaminase

### Perioperative management

PD has been often indicated for tumors of the pancreatic head region with obstructive jaundice. For such cases in our study, endoscopic retrograde biliary drainage was performed preoperatively by a gastroenterologist to reduce jaundice. Prophylactic antibiotics were administered at the time of surgery, and the same antibiotics were continued until the first postoperative day.

### Surgical procedure

All surgeries evaluated in this study were performed by experienced hepatobiliary pancreatic surgeons. The PD procedure performed at our institute was based on subtotal stomach-preserving PD, whereby the stomach antrum is resected to preserve most of the stomach. Reconstruction was performed using the modified Child method. After resection, anastomoses were constructed using a single jejunal loop that was brought through the resected ligament of Treitz. First, pancreaticojejunostomy was performed in an end-to-side manner. Subsequently, hepaticojejunostomy was performed in an end-to-side manner using a single layer of interrupted sutures. If the diameter of the remnant bile duct was small, then an internal or external drainage tube (6-Fr silicon tube) was placed at the anastomosis at the surgeon's discretion. Finally, with the patient in the retrocolic position, side-to-side gastrojejunostomy was performed approximately 35 cm downstream from the hepaticojejunostomy. Braun’s anastomosis was not added to the routine.

### Perioperative factors

Operative time, blood loss, blood transfusion, postoperative pancreatic fistula (POPF), bile leakage, and delayed gastric emptying (DGE) were extracted as perioperative factors. POPF was evaluated using the revised classification of the International Study Group of Pancreatic Fistula [11]; bile leakage was defined according to the guidelines of the International Study Group of Liver Surgery [12]. The DGE was evaluated using the classification of International Study Group of Pancreatic Surgery in 2007 [13].

### Outpatient management

Patients underwent postoperative follow-up assessments, including blood tests and imaging studies, at the outpatient clinic every four to six months. Patients with symptoms, such as fever, visited the emergency room or outpatient clinic. Blood tests, blood culture tests, and imaging studies were performed based on the discretion of the examining physician. When cholangitis was diagnosed, patients were often admitted to the general medicine ward and treated with intravenous antibacterial therapy. Pneumobilia was considered positive if the findings were found on imaging studies (computed tomography or ultrasonography) at postoperative follow-up.

### Statistical analysis

Continuous variables are expressed as the mean or median, standard deviation, and range. Categorical data were analyzed using either the chi-square test or Fisher’s exact test. Further, continuous data were analyzed using Student's t-test or Wilcoxon’s rank sum test for unpaired data, and paired t-test or Wilcoxon’s signed-rank test for paired data, as appropriate. A logistic regression analysis was performed in the multivariate assessment to determine independent risk factors. Baseline variables (P < 0.20 in the univariate analysis) were included in the multivariate analysis. Furthermore, preoperative biliary drainage [14] and postoperative pneumobilia [15], which have been previously reported as risk factors for severe complication after PD, were included in the multivariate model. All statistical analyses were performed using the JMP® Pro 14.2.0 software (SAS Institute Inc., Cary, NC, USA) and R version 4.0.0 (The R Foundation for Statistical Computing, Vienna, Austria). All analyses were two-tailed, and P < 0.05 was considered statistically significant.

## Results

The clinical characteristics of 176 patients are summarized in Table 2. The median age of these 176 patients was 70 years (range, 34–86 years); 63% were men; and the mean body mass index was 22.9 kg/m^2^. The most common disease associated with PD was pancreatic carcinoma (45%). Jaundice was detected in 47% of the patients, and preoperative biliary drainage, including prophylactic procedures, was performed in 59% of the patients. Preoperative cholangitis was detected in 20% of patients.

**Table 2:** Clinical characteristics of patients (*n* = 176)

Characteristic	Patients (*n*)	Percent
*Patient factors*		
Age, years		
Median (range)	70 (34–86)	
Interquartile range	63–76	
Sex		
Male	110	63
Female	66	37
Body mass index (kg/m^2^)		
Mean (SD)	22.9 (3.2)	
Range	15.4–35.3	
ASA-PS	27	15
I		
II	138	78
III	10	5.6
IV	1	0.5
Diabetes mellitus	28	16
Diagnosis		
Pancreatic carcinoma	79	45
Cholangiocarcinoma	47	27
Carcinoma of the ampulla of Vater	22	12
Others	28	16
Jaundice	82	47
Preoperative biliary drainage	104	59
Preoperative cholangitis	35	20
Postoperative cholangitis	19	11
*Operative factors*		
Operative time (min)		
Median (range)	451 (278–738)	
Interquartile range	381–497	
Blood loss (ml)		
Median (range)	594 (10–2450)	
Interquartile range	330–1195	
Blood transfusion	33	19
Width of remnant bile duct (mm)		
Median (range)	10 (4–40)	
Interquartile range	8–12	
Biliary stenting	94	53
*Postoperative factors*		
POPF		
Grade B or C	84	48
Bile leakage	4	2.2
DGE	9	5.1
Postoperative pneumobilia	132	76
Observation period (month)		
Median (range)	35 (11–115)	
Interquartile range	18–58	

*SD* standard deviation; *POPF* postoperative pancreatic fistula; *DGE* delayed gastric empty; *ASA-PS* American Society of Anesthesiologists Physical Status

Details of the PPDC are presented in Table 3. PPDC occurred in 19 of 176 (11%) patients; of these, nine had recurrent PPDC, resulting in a total of 36 episodes. Of the 19 patients who had PPDC, 14 (73%) experienced the first episode within two years after surgery. Based on the TG18, 21 of 36 (58%) cases were classified as mild and 15 of 36 (42%) cases were moderate; none of the cases were severe.

**Table 3:** Details of PPDC

Number of affected patients	19/176 (11%)
Total events of PPDC	36
Number of episodes of PPDC	Patients (n = 19)
1	10
2	3
3	5
5	1
Time from surgery to first onset (month)	Patients (n = 19)
≤ 24	14
> 24	5
Severity according to TG18	Total events (n = 36)
Mild	21 (58%)
Moderate	15 (42%)
Severe	0

*PPDC* post pancreaticoduodenectomy cholanitis, *TG18* Tokyo Guideline 2018

**Fig. 2:**
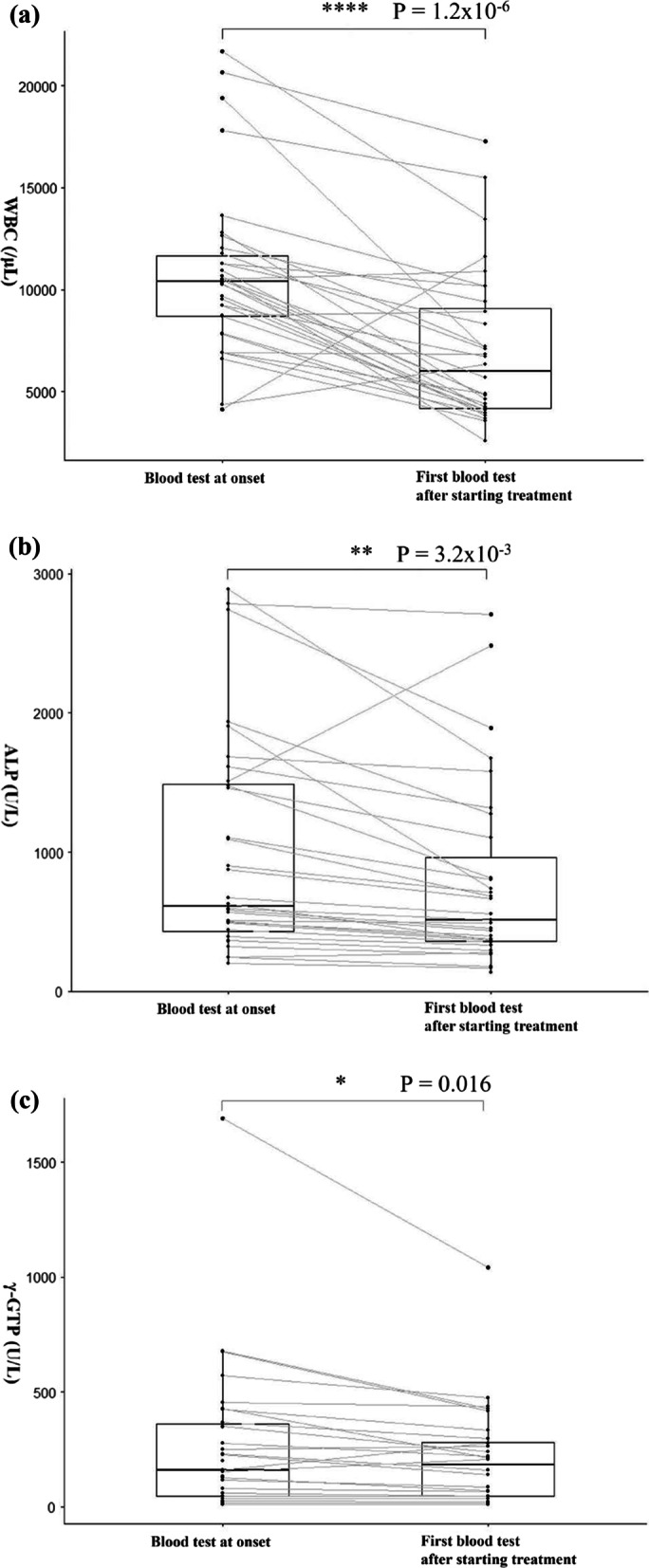
Changes in the blood test values of (**a**) WBC, (**b**) ALP, and (**c**) γ-GTP at the onset of post-PD cholangitis and after starting the initial treatment. Blood test results showed changes in the (**a**) white blood cell (WBC) counts, (**b**) alkaline phosphatase (ALP) levels, and (**c**) gamma-glutamyl transpeptidase (γ-GTP) values at the onset of post-pancreaticoduodenectomy cholangitis (PPDC) and after starting the initial treatment. For WBC counts, the median at the onset of PPDC and first blood test after starting treatment were 10,385 (range: 4120‒21,690) and 6000 (range: 2570‒17,260), respectively (**a**). The Wilcoxon signed-rank sum test for the two corresponding groups showed a statistically significant difference with P = 1.2 × 10^–6^ (**a**). For ALP level, the median at the onset of PPDC and first blood test after starting treatment were 614 (range: 204‒2890) and 516 (range: 135‒2706), respectively (**b**). A statistically significant difference was noted among the two corresponding groups, with P = 3.2 × 10^–3^ (**b**). For γ-GTP values, the median at the onset of PPDC and first blood test after starting treatment were 160 (range: 9‒1693) and 184 (range: 9‒1042), respectively (**c**). A statistically significant difference was noted among the two corresponding groups, with P = 0.016 (**c**)

The results of the blood tests showed changes in the white blood cell counts, alkaline phosphatase levels, and gamma-glutamyl transpeptidase values, before and after treatment was started. The first blood test was performed a median of three days later (range, 1–7 days) after the treatment began. Although the initial treatment was limited to antibiotics only, all of the blood tests for this cohort showed significant improvement after starting treatment (Fig. 2).

Details of the blood culture tests are presented in Table 4. Blood culture tests were performed for 24 episodes; of these 24 tests, 16 (67%) yielded positive results. Blood culture test results were positive for 10 of 12 (83%) episodes with mild disease and 6 of 12 (50%) episodes with moderate disease. *Escherichia coli* was the most frequently detected pathogen in the blood culture samples.

**Table 4:** PPDC patients blood culture results

*Blood cultures*		
Total number of inspections	24	
	Number of tests positive (%)	
Severity of PPDC		
Mild	10 /12 (83)	
Moderate	6 /12 (50)	
Detected bacterial species and the number of detections by severity		
	Number of detections by severity of PPDC	
Bacteria	*Mild*	*Moderate*
* Escherichia coli*	7	4
* Klebsiella pneumoniae*	2	2
* Aeromonas cavie*	2	0
* Enterococcus faecalis*	1	0
Others	4	2

*PPDC* post pancreaticoduodenectomy cholangitis

A diagnosis of postoperative cholangitis often resulted in hospitalization for intravenous antibacterial therapy for a median of seven days (range, 3–31 days). There was no difference in the duration of antibiotic administration based on the severity of PPDC (Table 5). No cases progressed to severe disease after the commencement of treatment. Sulbactam/ampicillin (SBT/ABPC) was the most frequently used initial antibiotic regimen, and antimicrobial agents were changed for 11 episodes based on susceptibility, indicated by blood culture test results (Table 5).

**Table 5:** Details of the treatment of PPDC patients

*Medication*		
	Duration of medication (day)	
All patients	7 (3–31)^a^	
Severity of PPDC		
Mild	7 (3–26)^a^	
Moderate	7 (5–31)^a^	
Medication for PPDC	Number of events	
Oral antibiotic treatment	6	
Intravenous antibiotic treatment	27	
Intravenous therapy followed by oral antibiotics	3	
*Antibacterial agents*		
First antibacterial agent administered	Number of events	
Sulbactam / ampicillin	21	
Ceftriaxone	5	
Levofloxacin (oral antibiotics)	6	
Others	4	
Cases with subsequent change of antibiotics	11	

Treatment for stenosis of hepaticojejunostomy		Patients (*n* = 4)
Patient no.	Number of events of PPDC	Treatment (severity of PPDC)
1	3	BD + SI (mild), SI (mild), BD + EL, SI (moderate), BD
2	2	BD + SI + EL (moderate), SI + EL (mild)
3	5	SI (moderate)
4	3	EO

*BD* Endoscopic baloon dilation; *SI* Endoscopic stent insertion; *EL* Endoscopic lithotripsy; *EO* Endoscopic observation only

^a^Data was presented as median (range)

An endoscopic examination was performed for a total of four patients with repeated cholangitis or suspected anastomotic stricture (Table 5). Three of these four patients required treatment for anastomotic stenosis. One of these patients underwent a total of five endoscopic procedures, including balloon dilation, stent insertion, and stone extraction. For one patient with three recurrent cholangitis episodes, the endoscopic examination demonstrated that the hepaticojejunostomy site was sufficiently open with no stenosis or stones. Therefore, the cause was thought to be simple reflux.

A univariate analysis of patients' preoperative, intraoperative, and postoperative characteristics was performed to determine their associations with the development of PPDC (Table 6). None of the characteristics was significantly associated with PPDC development. Postoperative pneumobilia was present in 75% of patients, but was not significantly associated with the occurrence of PPDC (Table 6).

**Table 6:** Predictors for the development of PPDC by univariate and multivariate analysis

Covariate	Category	PPDC		Univariate	Multivariate	
		Positive	Negative	P value	Odds ratio (95%CI)	P value
		*n* = 19	*n* = 157			
*Patient factors*						
Age (years)^a^		71.7 (10.6)	68.3 (9.90)	0.15	1.04 (0.99–1.10)	0.14
Sex	Male	14	96	0.28	1.86 (0.63–5.53)	0.26
	Female	5	61			
Body mass index (kg/m^2^)^a^		22.7 (2.8)	22.9 (3.2)	0.86		
ASA-PS	I or II	18	147	1.00		
	III or IV	1	10			
Diabetes mellitus	Positive	3	25	0.99		
	Negative	16	132			
Diagnosis						
Pancreatic carcinoma		9	70	0.77		
Cholangiocarcinoma		6	41			
Carcinoma of the ampulla of Vater		1	21			
Others		3	25			
Preoperative jaundice	Positive	9	73	0.94		
	Negative	10	84			
Preoperative biliary drainage	Positive	12	92	0.70	0.90 (0.32–2.58)	0.85
	Negative	7	65			
Preoperative cholangitis	Positive	5	30	0.54		
	Negative	14	127			
*Operative factors*						
Operative time (min)^a^		440 (94)	449 (81)	0.66		
Blood loss (ml)^b^		666 (28–1280)	588 (10–2450)	0.89		
Blood transfusion	Positive	2	31	0.53		
	Negative	17	126			
Width of remnant bile duct (mm)^a^		10.3 (3.2)	10.8 (4.3)	0.61		
Biliary stenting	Positive	11	83	0.67		
	Negative	8	74			
*Postoperative factors*						
POPF Grade B or C	Positive	8	76	0.63		
	Negative	11	81			
DGE	Positive	0	9	0.60		
	Negative	19	148			
Bile leakage	Positive	0	4	1.00		
	Negative	19	153			
Postoperative pneumobilia	Positive	17	115	0.14	1.67 (0.45–6.22)	0.45
	Negative	2	40			

*PPDC* post pancreaticoduodenectomy cholangitis, *CI* confidence interval, *ASA-PS* American Society of Anesthesiologists Physical Status

^a^Data were presented as mean (standard deviation)

^b^Data were presented as median (range)

In addition to age and sex, preoperative biliary drainage and postoperative pneumobilia were used as covariates in the multivariate analysis. The results of the multivariate analysis indicated that there were no significant predictive factors for PPDC development (Table 6).

## Discussion

This study investigated the clinical characteristics of patients with PPDC treated at our institution and evaluated their severity and treatment. The results demonstrated that most cases of PPDC occurred within 2 years of surgery and that half of the cases involved recurrence. Although the positive rate of blood culture tests was high, there were no cases of severe PPDC. Furthermore, there were no significant predictive factors for the development of PPDC.

Previous studies have reported that PPDC occurred in 6.7–36.1% of patients [15–19]. The details of PPDC treatment have been reported for 43 cases [15, 18, 19]. These studies showed that anastomotic stenosis was present in 16 cases (37%) and that many of them were treated endoscopically. Most cases without anastomotic stenosis were treated with antimicrobial agents. This study investigated the results of examinations for and treatment of PPDC. These results have not been previously described in detail.

Based on the clinical course of PPDC examined during this study, we made the following observations regarding the pathogenesis and optimal treatment of PPDC: first, the pathogenesis of PPDC was discussed in the absence of any suspicious anastomotic stenosis of the hepaticojejunostomy; although the proportion of positive blood culture test results was high, there were no severe cases, and the biliary enzyme values, which were high at the time of onset, improved only with short-term antibiotic treatment. Therefore, it has been speculated that the pathogenesis of PPDC is due to a temporary increase in biliary pressure following the regurgitation of digestive juices.

Stagnation of the flow of digestive juices in the jejunal loop can easily lead to reflux of digestive juices into the IHBD via anastomosis [18]. Shortening the jejunal loop to the minimum length necessary and adding the Braun's anastomosis may reduce pressure in the jejunal loop and prevent reflux of digestive fluids into the IHBD [20].

In contrast, impaired bile excretion in the IHBD is considered the pathogenesis for cases with anastomotic stenosis; moreover, this is also considered the pathogenesis for common acute cholangitis. Narrowing of the anastomosis of the hepaticojejunostomy may be accompanied by hepaticolithiasis, which may lead to a condition like common acute cholangitis. In this study, patients with recurrent cholangitis or suspected anastomotic stenosis were subjected to endoscopic examination and treatment that required multiple procedures; however, no severe cases were observed. Anastomotic stenosis may not be relieved by a single treatment; therefore, careful follow-up and the consideration of multiple possible procedures are recommended. Benign bile duct stenosis is the most common type of anastomotic stenosis; however, caution should be exercised in cases of possible malignant anastomotic recurrence.

PPDC severity was classified according to the TG18 severity criteria. There was no significant difference between mild and moderate diseases in terms of the proportion of positive blood culture test results and treatment duration. Classifications of mild PPDC and moderate PPDC according to the TG18 may be equivalent in terms of the treatment and clinical course.

The basis of treatment for PPDC, with or without anastomotic stenosis, is antibiotic administration. In this study, SBT/ABPC was often the first choice of antibacterial agent for PPDC and resulted in no cases of exacerbation. Additionally, SBT/ABPC can generally treat *E. coli*, which was frequently detected in blood culture samples in this study, and anaerobic bacteria such as *Bacteroides fragilis*. Therefore, it is conceivable that SBT/ABPC is a reasonable first choice for PPDC. Previous studies have shown that the overuse of SBT/ABPC has increased the resistant strains against SBT/ABPC in some areas; therefore, blood culture tests should be performed before the use of antibiotics to confirm the causative organism and drug sensitivity [10, 21]. For severe cases, we concur with the TG18 regarding the proposed use of metronidazole and clindamycin to treat *B. fragilis* with cholangitis after hepaticojejunostomy [10].

In this study, a short course of antimicrobial therapy resulted in clinical improvement even when Gram-negative bacilli, such as *E. coli*, were detected in blood culture samples. It has been reported that during the treatment of common acute cholangitis with Gram-negative bacilli, the antibiotic administration period could be shortened to less than two weeks, which is the conventional administration period if the source of infection is controlled, and we believe that this applies to the administration period for PPDC [22].

Regarding the route of administration, most patients in this study were hospitalized and treated with intravenous infusion. The TG18 indicated that oral antimicrobial therapy is also acceptable for common acute cholangitis depending on the patient’s ability of oral consumption [10]. For mild PPDC cases, follow-up with oral antibiotics in an outpatient clinic may be considered.

One limitation of this study was that most of the cases were observed for more than one year but less than two years, which is an insufficient postoperative evaluation period. Additionally, we did not encounter any severe cases of PPDC. Moreover, this was a retrospective study performed at a single institution. To clarify the pathophysiology of PPDC, identify its possible predictors, and develop its disease-specific therapeutic recommendations, we believe prospective future studies are essential.

## Conclusion

Most cases of PPDC occurred within two years of surgery, and half of the cases demonstrated recurrence. While the positive rate of blood culture tests was high, no severe cases were observed. The pathogenesis of PPDC may involve increased temporal pressure in the IHBD, which improves relatively promptly with antimicrobial treatment alone. Endoscopic examination should be considered for cases of recurrent cholangitis, which may be caused by an anastomotic stenosis.

## Data Availability

The datasets analyzed during the current study are available from the corresponding author on reasonable request.

## Abbreviations

PD: Pancreaticoduodenectomy
PPDC: Post pancreaticoduodenectomy cholangitis
IHBD: Intrahepatic bile duct
TG: Tokyo Guidelines
SBT/ABPC: Sulbactam/Ampicillin
